# Vital Function in Dentine

**Published:** 1866-11

**Authors:** Wm. M. Rogers

**Affiliations:** Shelbyville, Ky.


### VITAL FUNCTION IN DENTINE.

BY DR. WM. M. ROGERS, SHELBYVILLE, KY.

Much discussion has been held upon the character of the functions performed in the teeth; many important questions have been raised, debated, and abandoned to their native darkness. We grope in doubt and perplexity—we suggest vascularity, we turn to the fact of cellular circulation, as embraced in the idea of *exosmosis* and *endosmosis*; but foiled in the search for palpable evidences, we relapse into the consciousness that all is but vanity and vexation of spirit.

Talent and energy enough have been devoted to this subject to have worked greater results in almost any other field of knowledge. Yet, if these results have not been conclusive, they are still important, and reflect the highest honor upon the student. Light, light, more light is needed. Without it dental practice must be largely empirical.

In order to treat disease with a clear degree of intelligence, we must be familiar with the organism to which attention is given. If we cannot demonstrate an anatomical structure, what shall we say of its physiology; and how shall we comprehend its pathological conditions?

I apprehend that all treatment must be based either upon experience or upon a scientific comprehension of the conditions to be met. The first can never in itself be completely satisfactory, even in its success, to our intelligence; the other must be predicated upon ascertained truth, or upon principles legitimately educed from it.

Accordingly as we admit or deny the agency of vital action in the phenomena exhibited in the teeth, so must we be confined to the beaten round of empiricism, or diverge into fields of discovery and progress; hence the subject is one of practical importance, and should be squarely met and decided for himself by every practitioner of dentistry.

The basis idea of this paper is the unity and continuity of the *plan* of God's creation—unity and continuity, whether considered as a long line of connecting links, or as an infinitude of groups and families, overspreading and fusing into each other in geometric circles.

Every character of reasoning must have an element of fact upon which the foundations are placed. We must rise from experience to law; and then, applying the law to the arranged facts, we may trace connections, nay, we may venture to supply them, just as a rational man would supply a lost crank to complete the connections of machinery.

Raise from their vulcanized beds the extinct species of the vegetable kingdom, and you find a vacant page upon the botanist's book for them. Exhume the giant bones of the mastodon, and he marches to his unfilled place in the ranks of creation, and passes the number "one, two," like a veteran as he is.

One of the difficulties attendant upon investigations of the dental organs arises, I apprehend, from imperfect nomenclature. Our nomenclature, at the first an offspring of our study, becomes at length the mould, and finally the very concretion of our received ideas.

If we say that dentine is susceptible of inflammation, we are told that some of the symptoms are not exhibited in the case which we so diagnose. If we assert that it is vascular, a demonstration is required, and we fail. In the one place we must claim an inflammation modified by the character of the organ in which it exists; in the other a vascularity so modified and obscured as to be but a pseudo-vascularity. Or, if you will, let me yield the point, and predicate a cellular arrangement. In fine, we must claim that the phenomena of vital and morbific action in dentine are just such as the character of the structure will admit, and not otherwise.

If we would vindicate the *harmony* of nature, we should claim another position, that is, that in a changed condition of organism by nature from her well defined plan of execution, we are not to look for a change of *law or plan*, but for a modification in adaptation to the purposes to be subserved, and then only so far as the purpose may demand. We can admit of no caprice—no mere expedient of a puzzled contriver.

The various modifications of the cutis and mucous membrane are illustrations of this position. In analogy with these we might be permitted to place the enamel, with its subponed sensorial tissue; and, under perfect normal development, its super-consolidated pellicle.

A striking illustration of the idea may also be traced in the morphology of plants. Here the seed, the embryo of the future plant—the evolving plumule—the multiplicity of succeeding terminal and auxillary leaf-buds—the metamorphosed fruit-bud—the bracts and sipals, the stamens and pistils of the flower, all are *modifications* of the chosen type, *the leaf*, changed in adaptation to the purpose to be accomplished. One step only in the stage of metamorphosis might have indicated a simple contriver and an obedience to expediency; but the continuity of modification, in unity of expression, clearly indicates a line of legal sequences, in which are shadowed the breadth and compass of the architect moving on to his designs in unity of purpose and harmony of conception. Here let us note the fact that in all this change of structure there is no point in which the vital functions are superceded, or from which the vital fluid is excluded. The limits of the one are the *ultima thule* of the other.

How many thousands of different species of plants does the vegetable kingdom present? How long might we turn the microscope to another and another of these, in the vain effort to detect nature in the act of circulation? Shall we conclude, in the absence of demonstration, that we must reject the fact? But in some of the water plants, the circulation in the cells of the leaves is at last discerned and the question is at rest.

If we cannot detect a circulation in dentine, have we not the strongest reasons to assume the fact? Let it be never so feeble, and never so subtle, when once admitted, everything arranges about it in harmony. If one character of circulation is impossible, then another is demanded, lying far out, it may be, upon the extremity of vital forces; but none the less are organism, life, function and nutrition, to be held as correlated and inseparable facts. We may here refer to a fact within the knowledge of every horticulturist, illustrative of the law of adaptation in changes of living organism. In the operations of budding and grafting, a degree of congeniality between the stock and the inserted bud or graft is essential. As men do not gather grapes of thorns, nor figs of thistles, neither do they transfer the grape upon the thorn, nor the fig-tree upon the thistle.

The pear is inserted upon the quince, and, under favorable circumstances, it flourishes for an indefinite time. It occurs that there are varieties of the pear that refuse this alliance, or hold it feebly. For such an intervening stock must be supplied. Place the sickle upon the quince, and it grows feebly, and at last succumbs; intervene the Duchess, and it grows in health and vigor. I cannot admit that this illustration is impertinent, as being an interference with nature's own courses, we but put the witness to the test of a cross-examination, and, true to her proclivities, she answers in the line of law, and demands an adaptation through which the life-forces may be exhibited.

The suggestions do not stop here; but I have pursued them as far as I designed.

In the osseous structures of the teeth, we have, first, *cementum*, the intervening modification between periosteum and dentine; then, *dentine*, and *enamel*. Chemical analysis gives us, in these substances, a varied amount, relatively, of organic and inorganic material, cementum having the largest proportion of organic substance, next to which are dentine and enamel in order. With reference to the inorganic constituents, the converse is true. In microscopical examinations these substances recede from the more palpable evidences of vascular structure in the same direction.

There is a comparative conformity of the pulp to the external periphery of the tooth. Is this a coincidence, or is it in some way founded on the reason of things? If the teeth are mere mechanical structures, would not a solid crown have been indicated? Why this little *intero-organism*, and this conformity? Look at the compact structures to be nourished. They must, of necessity, from this fact, be placed close around the base of supply; the faintness of their vital organism demands it.

The pabulum of the teeth must be borne through their substance by a solvent. In normal conditions this solvent yields its treasures, it may be, to the assimilating functions, and hence the consolidation of dentine during the advance of life. It may take, in solution, the effete matter presented, or it may reduce the solidity of structure, and thus become correlative with chemical action in the production of decay. It is entirely comprehensible that a given cause may be sufficient for the production of an effect, and yet not be the only cause involved, but that there may be other and independent causes concurrent with and conducing the result, and yet not essential.

Every dentist is familiar with a class of long teeth, in which the pulp lies deep, a class that tolerates larger fillings, perhaps, than any other.

They are usually yellowish in color, and below the medium in density.

Is not vital action always feeble in such? Again, we have seen a class, short, compact, and very dense in structure. They are usually blotched with yellow in the enamel, and highly sensitive at the juncture of the enamel and dentine. Do these teeth not exhibit an instance of the strangulation of the nutrient organism in this super-consolidated enamel? I

am inclined to think so; but I design the questions as simply suggestive.

Again, are not all those cases of the consolidation of dentine after filing and polishing, and after the near approach to the pulp in filling, the result of the faint remedial effort of *organism* to protect the citadel of its safety? Why the surcharged condition of the dental vital fluid with the constituents of dentine, resulting in *spiculæ* and nodules of secondary dentine within the pulp cavity, and in hypertrophy of the fangs? Have we here a supply in excess of demand, or is access to the point of demand no longer attainable, and a calciferous congestion and deposit the result?

We have seen dental structures by abrasion of the coronal faces, followed by deposit of secondary dentine, thrown out over the retreating pulp. This deposit is very dense, and is blackened and glassy in appearance. Will the principles involved in the foregoing remarks account for this? Have we not another instance of strangulation, and a sort of atrophy, in which the organic constituent is represented in the carbon of its composition, left as the *debris* of the retiring pulp?

Bind a ligature around an orange tree, or a silver-leaf poplar, and in a few years an enlargement occurs just above the ligature. It will often appear, after grafting trees, that the scion in progress of growth enlarges into a monstrosity, just at the point of juncture with the stock. Here we have a manifest arrest of the wood-producing pabulum, resulting in hypertrophic deposits. Are not the cases of hypertrophy of the fangs and deposits of dentine in the pulp-cavities of the teeth referable to the principles involved in this condition of vegetable pathology, if we may so designate it?

I have rapidly scanned my subject; it will be seen that it might have been indefinitely elaborated; but I have preferred to touch the points involved, and to indicate what I suppose to be their bearings, leaving the intelligent dentist to pursue them if he may think them worthy of such attention. I have not attempted to indicate a hypothesis of structural arrangement, but to support the theory of vital function in dentine.

If there be those who have already planted their feet upon the platform of "*a priori*" reasoning, and have leaped the chasm to the solid facts of demonstrated structure, to such these remarks, having been anticipated, are useless. But are not all such aware that too little attention is given to the idea of *vital* phenomena in the teeth? Can there be progress in any other direction? Can we admit that the Creator finds in these organs a successful barrier to the transcendent *plan* unfolded in us, above us, below us, and around us, in such wondrous harmony and unity? Is there a necessity above God? Here I would suspend the question involved in this essay.

